# MagGami: An Electromagnetic Origami Actuator With High Stiffness‐Switching Density for Lightweight Joint‐Space Wearable Haptics

**DOI:** 10.1002/advs.78363

**Published:** 2026-10-20

**Authors:** Tianhao Jin, Zirui Niu, Qingyun Tang, Zemin Wang, Yu Qiao, Guocai Yao, Yong Zhou, Boxue Shan, Yan Zhang, Dangxiao Wang

**Affiliations:** ^1^ State Key Laboratory of Virtual Reality Technology and Systems Beihang University Beijing China; ^2^ School of Mechanical Engineering and Automation Beihang University Beijing China; ^3^ Beijing Academy of Artificial Intelligence Beijing China; ^4^ Linkerbot Beijing Technology Co., Ltd Beijing China

**Keywords:** joint‐space feedback, magnetic force, origami robotics, wearable haptics

## Abstract

Kinesthetic feedback gloves play a central role in immersive virtual and augmented reality (VR/AR) by enabling the perception of object shape, stiffness, and spatial constraints. However, key challenges remain: 1) achieving a large stiffness contrast between free‐space and constrained‐space states, 2) enabling independent actuation on finger joints, and 3) maintaining lightweight, low‐power wearable operation. Here, we introduce MagGami, a miniaturized origami‐based variable‐stiffness actuator weighing 1.2 g for distributed joint‐space integration. By combining origami geometric interlocking with an electromagnetic bistable mechanism, it can be triggered by transient current pulses at the flattened 0° configuration of the origami mechanism. MagGami switches from a freely compliant state to a self‐locked high‐resistance state with a mean locking response time of 102.8 ms, while requiring no coil power to hold the locked state. The mean device‐level locked‐to‐free effective rotational‐stiffness ratio is 795.46, corresponding to a module‐level stiffness‐switching density of 6.63 × 10^5^ kg^−^^1^, and the specified switching cycle consumes 444 mJ. MagGami uses distributed joint‐space actuation to deliver lightweight, independently addressable binary constraint cues at individual finger joints, thereby enhancing the perception of virtual spatial constraints and high‐resistance contact in VR/AR interactions. This work demonstrates a compact and energy‐efficient strategy for next‐generation wearable haptic interfaces.

## Graphical abstract

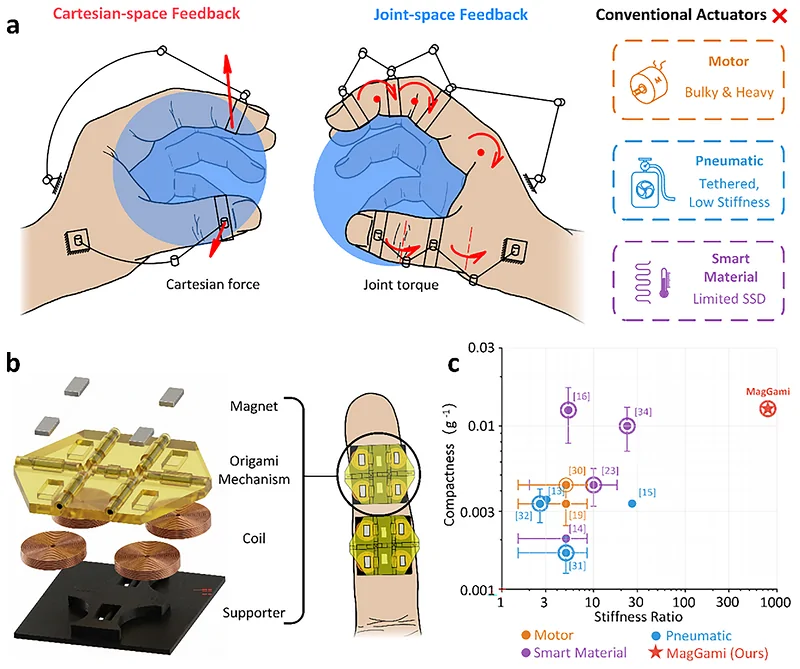

## 1 Introduction

High‐fidelity haptic feedback has become a key enabling technology for deeply immersive experiences in virtual and augmented reality (VR/AR), teleoperation, and advanced human‐computer interaction (HCI) [1, 2, 3, 4, 5]. As a critical bridge between digital twins and physical perception, haptic gloves allow users to perceive virtual boundaries, stiffness differences, and spatial constraint cues through kinesthetic feedback [6, 7, 8]. Such feedback is essential for enhancing embodiment, interaction realism, and task performance in immersive environments [9, 10, 11]. These demands place stringent requirements on device compactness, output force, and dynamic response.

Kinesthetic feedback gloves based on a variety of working principles have been developed, including motor‐driven systems [12], pneumatic systems [13], and smart‐material‐based systems (Figure 1a) [14, 15, 16, 17]. Most existing mature commercial and research prototypes still rely on a Cartesian‐space mapping paradigm, in which actuation sources drive dorsal exoskeletons or cable systems to generate a global pulling force only at the fingertip [18, 19, 20, 21, 22, 23]. Such a design reduces the three‐degree‐of‐freedom bending behavior of a single finger to an underactuated whole‐finger system [24], making it difficult to faithfully render complex local spatial geometries beyond the fingertip during human‐computer interaction. By contrast, a joint‐space mapping paradigm, in which force feedback is applied directly at each finger joint, can provide localized multipoint constraints that better match human finger biomechanics [25], thereby enabling more faithful rendering of the local geometric features and spatial contours of virtual objects [26, 27].

**FIGURE 1:**
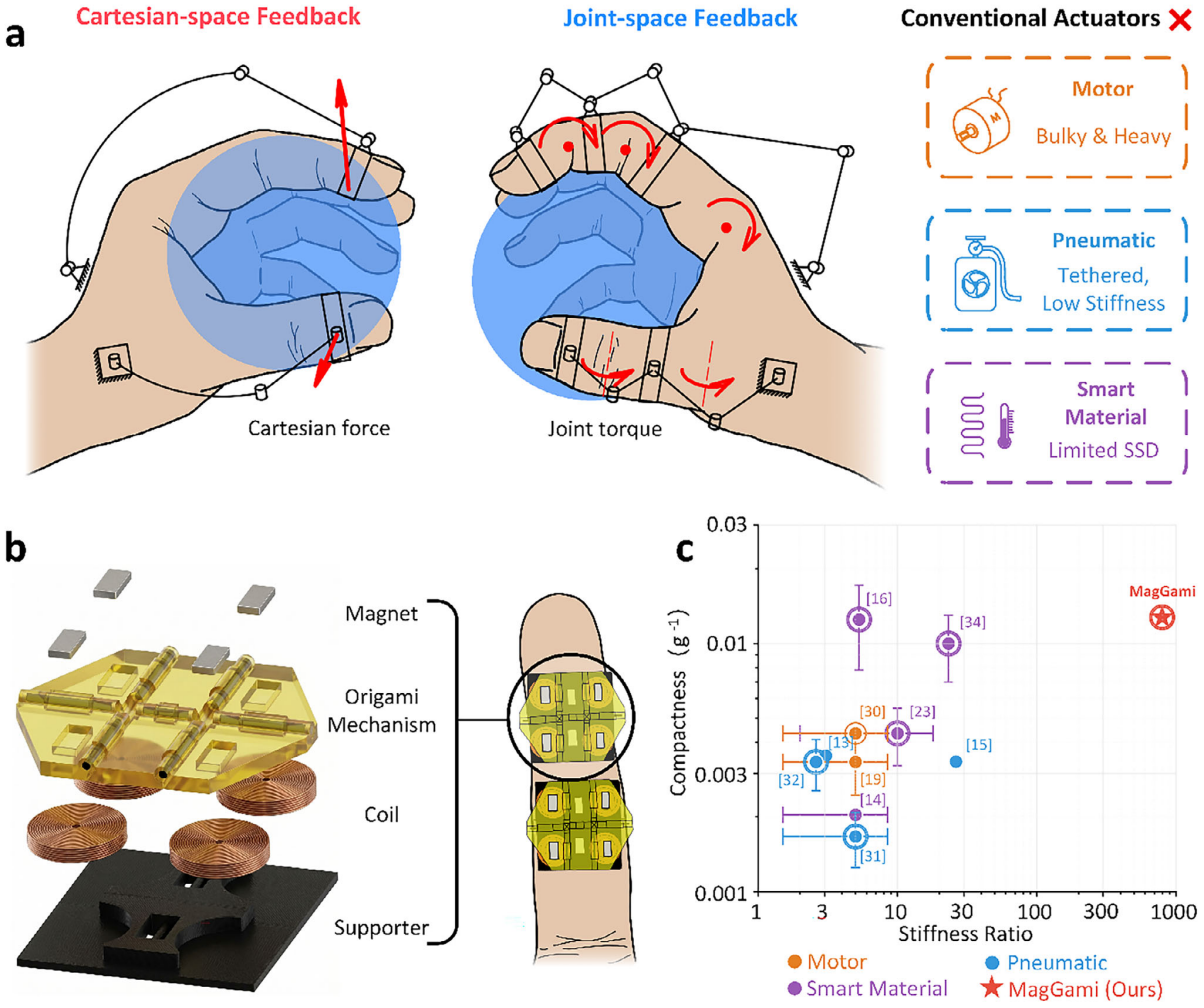
Concept of the MagGami‐based joint‐space haptic glove. a) Comparison between Cartesian‐space fingertip force feedback and distributed joint‐space torque feedback, highlighting the limitations of representative motor‐driven, pneumatic, and smart material‐based haptic actuators. b) Exploded structural design of the MagGami actuator and its finger‐joint integration. c) System‐level design‐space comparison in terms of stiffness ratio and compactness, defined as the inverse total mass of the complete wearable system. Colors denote actuator types; pale markers and error bars indicate estimated ranges. Markers without an outer ring, with a thin outer ring, and with a thick outer ring indicate 1, 2, and 3 feedback DoFs per finger, respectively.

However, delivering high‐performance multi‐DOF force feedback in joint space requires simultaneously high stiffness contrast, lightweight integration, and a large number of independently addressable feedback nodes (Figure 1c; Table S1) [28]. Each feedback node should combine low resistance during free motion with a pronounced locked‐state constraint while adding minimal mass. The stiffness ratio characterizes the contrast between these states but does not account for mass. Torque density measures absolute torque capacity per unit mass and is one form of output capability per unit mass, which may also be expressed in terms of force, work, or power. We therefore define module‐level stiffness‐switching density (SSD) as the locked‐to‐free effective rotational‐stiffness ratio divided by the mass of one actuator module. SSD captures the stiffness contrast obtained for the added actuator mass, making it useful for distributed wearable haptics, where worn mass accumulates with the number of nodes. The broader impedance variation range can include stiffness, damping, and inertia; the quasi‐static SSD used here characterizes only the contrast in effective rotational stiffness. We therefore report SSD together with absolute resisted‐torque capacity. Meeting these combined requirements remains challenging for conventional actuation technologies. Conventional power sources, such as motor‐driven mechanisms, are bulky and heavy, both in the actuator itself and in the associated transmission components [29, 30]. Pneumatic actuation offers lower weight by comparison, but pneumatic tubing systems often suffer from tethered systems and limited stiffness contrast, making them inadequate for rendering highly rigid objects [31, 32]. Other systems based on emerging materials, such as SMAs, also face major limitations, including slow response, continuous heat generation, and non‐negligible parasitic resistance in free space [33, 34].

Notably, origami mechanisms offer a highly promising strategy for addressing this challenge. Owing to their scalability and recent advances in microscale 3D printing, origami‐based metamaterials can achieve remarkable compactness and precision, opening new opportunities for ultra‐lightweight wearable devices [35, 36, 37, 38, 39, 40]. Recently, several groups have explored origami‐based haptic interfaces in various form factors, including the compact multi‐degree‐of‐freedom finger‐pad force device by Winston et al. [38], the 18‐g haptic feedback ring with integrated three‐axis force sensing by Kang et al. [39], and the hand‐held and foot‐stepping interfaces for active stiffness perception using curved‐origami designs by Zhang et al. [41]. Beyond haptic interfaces, Liu et al. demonstrated a general origami‐actuation paradigm in which an external driving source supplies the actuation energy, while the origami‐based body governs the resulting motion and mechanical performance; in their pneumatic implementation, small‐strain folding of a semi‐rigid thermoplastic polyurethane body suppresses in‐plane stretching, guides axial motion, and improves force transmission [42]. Recent studies have further extended origami actuation to electromagnetic triggering, demonstrating its potential for lightweight and fast‐response operation [43, 44]. While bistable electromagnetic actuation has been demonstrated in other origami systems [45, 46], its integration into distributed, low‐profile actuators for wearable joint‐space haptic feedback has not yet been realized.

Here, we present MagGami, a miniature origami‐based electromagnetically switchable variable‐stiffness actuator for joint‐space kinesthetic feedback (Figure 1b). The main contributions are threefold. First, we combine thick‐panel origami interlocking with pulse‐driven electromagnetic switching to realize a joint‐mounted actuator weighing 1.2 g and measuring 2.5 mm in thickness, with a mean locking response time of 102.8 ms. Second, we experimentally demonstrate a mean device‐level locked‐to‐free effective rotational‐stiffness ratio of 795.46 and a module‐level SSD of 6.63×10^5 kg^−^^1^. The mean maximum externally applied torque resisted by an individual actuator before structural failure is 76.25 N·mm (n = 5 actuators). Locked‐state holding requires zero coil power, whereas the specified switching cycle consumes 444 mJ per actuator. Third, we integrate nine independently addressable actuators into a joint‐space haptic glove and evaluate it with 10 participants, demonstrating higher perceived resistance in the constrained condition than in the free condition and reliable discrimination of single‐joint and multi‐joint constraint patterns.

## 2 Results

### 2.1 Design of the Interlocking Origami Actuator

The essence of kinesthetic feedback lies in the mapping between actuator states and two mechanical state spaces (Figure 2a). In free space, the system must maintain exceptionally high mechanical compliance so that it can unobtrusively accommodate the natural flexion and extension of the finger (stage i). In constrained space, by contrast, when encountering a physical boundary, the system must instantaneously generate a substantial resistive torque to prevent further joint flexion (stage ii). Thus, the central design challenge is to realize efficient and reversible switching between free and constrained mechanical states within an ultracompact wearable form factor.

**FIGURE 2:**
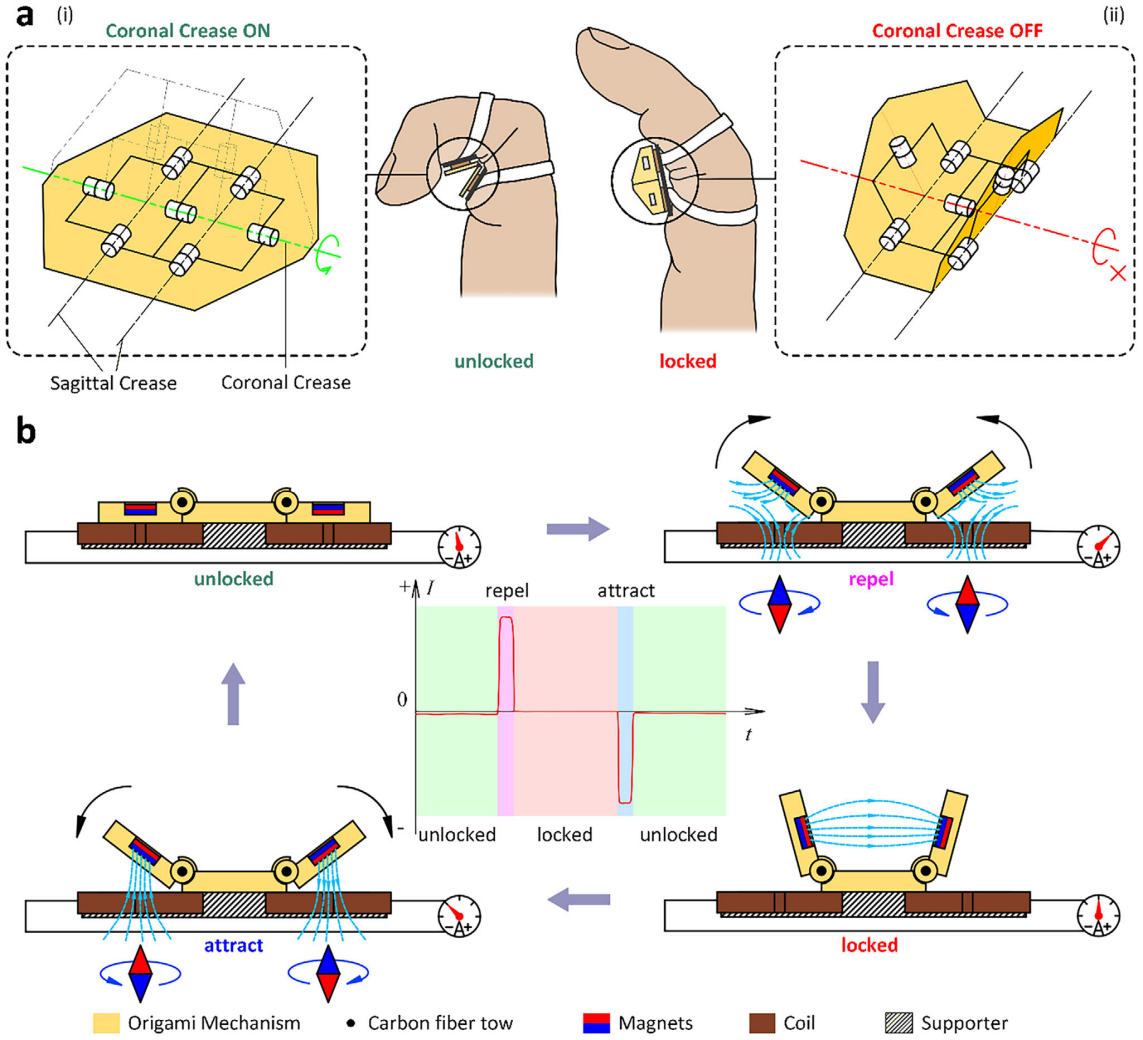
Structural design and bistable switching principle of the MagGami actuator. a) Origami kinematic interlocking mechanism and its finger‐joint states. In the unlocked state, a weak bias current maintains magnetic adhesion between the side panels and the base, allowing the coronal crease to follow natural joint flexion. In the locked state, folding of the sagittal creases induces geometric interlocking and restricts further joint motion. b) Electromagnetic switching cycle of MagGami. Transient repulsive and attractive current pulses drive reversible transitions between the unlocked and locked states. After locking, the coil is de‐energized, and the constrained state is maintained by magnet–magnet attraction and origami interlocking without static coil power.

To address this challenge, the MagGami actuator is designed on the basis of the kinematic interlocking behavior of orthogonal creases in thick‐panel origami. In an ideal planar origami model, when the central primary crease (the coronal crease) remains fully flattened, corresponding to the 0° kinematic singularity, the two lateral secondary creases (the sagittal creases) still retain their folding degrees of freedom. However, once either sagittal crease bends, the spatial configuration of the coronal crease becomes geometrically locked by the boundary constraints, causing an abrupt stiffness transition in the overall structure and completely eliminating its degree of freedom in the bending direction of the coronal crease. Exploiting this geometric feature, we precisely align the coronal crease of the actuator with the rotational axis of the human interphalangeal joint. As a result, in the free state (stage i), the sagittal creases remain flattened, allowing the coronal crease to exhibit excellent compliance so that the actuator can naturally follow finger flexion. When force feedback is required, the sagittal creases rapidly fold inward, the coronal crease becomes locked, and the mechanism transitions into a high‐stiffness constrained configuration that restricts further joint motion.

To achieve the extreme compactness required for independent single‐finger wearability, we performed extensive optimization at both the materials‐engineering and micromechanical‐layout levels (Figure S1). First, the rigid origami panels were monolithically fabricated from a high‐modulus photosensitive resin using high‐resolution 3D printing, thereby providing robust bending resistance in the locked state. Second, to overcome the excessive bulk associated with conventional pin‐joint hinges, we introduced miniature carbon‐fiber bundles with a diameter of only 0.3 mm as rotational hinge shafts. Owing to the exceptionally high shear strength and lightweight nature of carbon fiber, these microscale shafts maintain outstanding mechanical reliability under the large shear stresses generated by pronounced finger‐joint bending, thereby avoiding fracture failure. More importantly, we adopted a single‐sided eccentric shaft arrangement in the hinge design, such that the rotational axis was fully embedded within the physical boundary on one side of the panel. This spatial configuration reduces the Z‐axis thickness of the origami mechanism itself to only 1.2 mm. As a result, the actuator conforms more naturally to the physiological curvature of the finger joint while avoiding physical interference with unrestricted joint motion. These structural considerations at the microscale establish the physical basis for the subsequently achieved ultrahigh SSD.

### 2.2 Operating Principle and Static Characteristics

Beyond its geometric interlocking design, a second key innovation of MagGami is the incorporation of a bistable electromagnetic actuation mechanism, which breaks the trade‐off in conventional force‐feedback gloves between high‐impedance constraint and high static power consumption (Figure 2b).

State switching in MagGami is enabled by non‐contact magnetic coupling between the base coil and the permanent magnets embedded in the origami side panels. In the initial steady state corresponding to virtual free space (unlocked stage), only a minimal resting current is supplied to the coil. The weak magnetic field thereby generated produces a constant attractive force with the permanent magnets, ensuring that the side panels remain tightly attached to the base during high‐frequency, large‐amplitude finger motion. As a result, the origami structure is maintained in a fully flattened configuration, allowing unrestricted joint flexion and extension.

Upon detection of a spatial constraint event, such as contact with a virtual boundary, the actuator rapidly transitions to the locked state (repel stage). In this process, the microcontroller (MCU) delivers a transient high‐energy pulse current to the coil (repel pulse). The pulse generates a strong magnetic field with the same polarity as the permanent magnets, producing sufficient electromagnetic repulsion to overcome the initial holding attraction and drive the two side panels to rotate upward along the sagittal creases until reaching the designed mechanical range‐of‐motion limiter.

The most important mechanical and energetic features of the system are manifested in the post‐locking steady state. Once the side panels are fully deployed (locked stage), the embedded permanent magnets on both sides become firmly coupled through strong short‐range attraction between opposite poles. The coil is then completely de‐energized. Through this self‐locking mechanism, governed solely by internal magnetic attraction and origami‐enabled geometric interference, the actuator can stably maintain a high‐impedance restricted micro‐ flexion state even under substantial externally applied bending torque at the finger joint.

Importantly, this constrained state is sustained with near‐zero static power consumption, fundamentally mitigating the thermal dissipation and power‐load issues associated with prolonged force‐feedback operation. When the virtual interaction terminates and the system needs to return to the free state, a reverse transient pulse current is applied to the coil (attract stage). The resulting reverse electromagnetic attraction overcomes the magnetic coupling between the two permanent magnets and rapidly restores the side panels to the initial flattened state. The coil current then immediately returns to the weak resting level, completing a full bistable closed‐loop interaction cycle (Figure 2b). This control strategy, featuring transient pulse‐driven switching, zero‐power rigid self‐locking, and micro‐current resting maintenance, endows MagGami with exceptional energy efficiency and provides a promising hardware route toward long‐endurance fully actuated wearable haptic systems.

At the single‐node level, MagGami is intentionally designed as a binary high‐contrast constraint module rather than as a continuously tunable material‐stiffness display. This design choice is central to the proposed wearable paradigm: each module remains mechanically unobtrusive during free motion and becomes a self‐locked local joint constraint when a virtual boundary is encountered. When such modules are distributed across multiple anatomical joints, the glove does not emulate stiffness by arbitrarily changing the activation location; instead, it conveys where finger motion is locally restricted, thereby providing spatially organized joint‐space cues that enhance the perception of virtual geometric constraints and high‐resistance contact.

### 2.3 Actuator Parameters and Performance Evaluation

The mechanical performance of the actuator determines the fidelity of haptic interaction. To establish design criteria for locking initiation, steady‐state retention, and state switching, MagGami was theoretically modeled and experimentally validated from two perspectives, namely kinematic constraint and magnetic dipole interaction (Figure 3).

**FIGURE 3:**
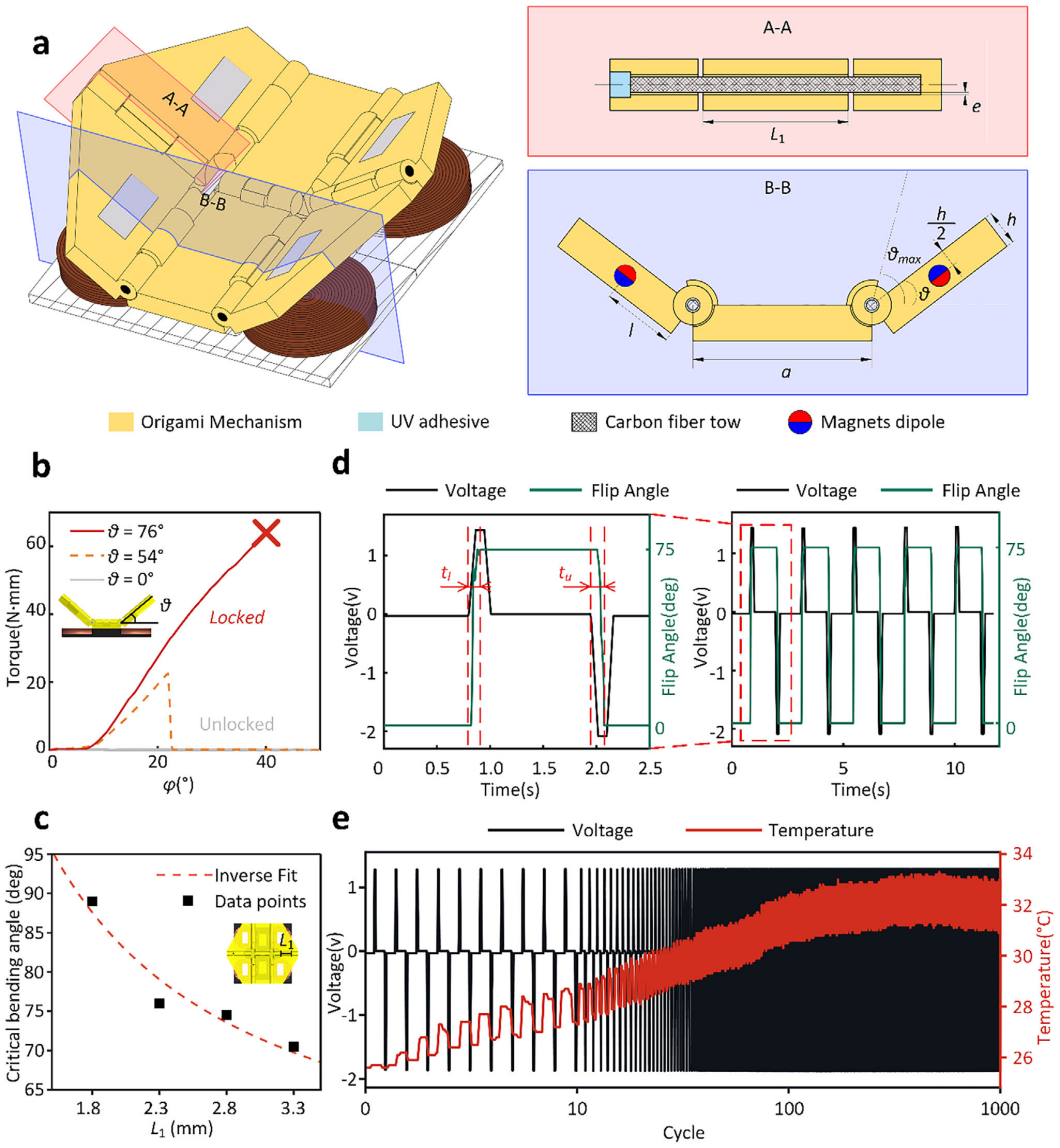
Mechanical characterization and dynamic performance of the MagGami actuator. a) Key geometric and electromagnetic design parameters of the origami interlocking actuator. b) Representative torque–angle responses of the MagGami actuator under different stop‐limited flip angles. c) Relationship between the middle segment length L_1_ and the critical bending angle for mechanical locking. d) Applied voltage and side‐panel angle of a representative actuator during one annotated locking–release cycle and five consecutive switching cycles. The marked timing points define the locking and releasing response times (*t_l_*, locking; *t_u_*, releasing). e) Cyclic actuation test showing the applied voltage pulses and temperature evolution over 1,000 cycles under a 2.3‐s operating cycle, with the maximum temperature remaining at 33.1°C.

First, the interlocking capability of the actuator is fundamentally limited by the spatial kinematics of the origami mechanism itself (Figure 3a). Under ideal conditions, that is, with perfectly rigid materials and zero clearance at the hinge holes, the mechanism approaches a rigid state in three‐dimensional space when the bending angle of the two side panels about the sagittal creases reaches any prescribed flip‐up angle θ, and bending of the coronal crease is then completely constrained. In the practical mechanism, however, a small radial clearance *e* exists between the shaft and the hinge holes, introducing a certain wobble tolerance that still allows bending of the central crease when θ is small. We define the critical state at which dynamic interference occurs in the coronal crease as the locking threshold. Through geometric derivation, the critical bending angle θ_cri_ can be expressed as:

(1) $$\begin{array}{c} \;\theta_{\mathrm{cri}}=\frac{2e}{kL_{1}} \end{array}$$

where *L*_1_ is the hole length of the middle segment in the side panel, and *k* is a proportionality factor associated with the panel geometry and material elasticity. This model reveals how geometric tolerance regulates the locking range: decreasing the clearance e or increasing the panel length *L*_1_ can significantly reduce θ_cri_, thereby enabling the mechanism to enter the locked state at smaller bending angles. Experimental results confirm this prediction (Figure 3c). By measuring the critical bending angle while varying *L*_1_, we verified that increasing *L*_1_ leads to a decrease in θ_cri_. Considering both the need to avoid motion interference with the width of the human finger and to minimize energy consumption, we selected *L*_1_ =  2.8 mm and *e*  =  0.05 mm. With these parameters, the actuator entered the mechanically interlocked regime when θ ≥ 72°. The mechanical stop was therefore designed to limit side‐panel deployment to θ_*max*_  =  75°, ensuring reliable geometric interlocking while balancing magnet–magnet retention and reversible coil‐driven release.

Second, the steady‐state retention capability after locking originates from magnetic‐force coupling between the embedded permanent magnets. Once in the locked state, the permanent magnets embedded in the two side panels generate an attractive dipole interaction. Considering the finite thickness of the origami mechanism, the magnet–magnet distance *d* can be written as:

(2) $$\begin{array}{c} d=a+2lcos\theta +hsin\theta \end{array}$$

where *a* is the distance between the two rotation axes, *l* is the distance from the magnet center to the rotation axis, *h* is the thickness of the origami mechanism, and θ is the flip angle of the side panel. Based on the magnetic dipole model (Figure S2; see Note S1 for the detailed derivation), the resulting self‐locking torque τ_lock_ can be expressed as:

(3) $$\begin{array}{c} \begin{array}{c} \;\tau_{\mathrm{lock}}=-\frac{\mu_{0}m^{2}}{4\pi }\left[\frac{3\left(1+\mathrm{si}n^{2}\theta \right)\left(2l\sin\theta -h\cos\theta \right)}{d^{4}}+\frac{2\sin\theta \cos\theta }{d^{3}}\right] \end{array} \end{array}$$

where *m* is the magnetic dipole moment of the permanent magnet. This self‐locking torque is essential for maintaining the high‐impedance constrained state of MagGami at zero static power consumption. At the deployed configuration defined by the mechanical stop, this self‐locking torque maintains the constrained state without continuous coil power, while release is achieved by the reverse current pulse described below.

The dynamic response during state switching is governed by transient electromagnetic interaction between the base coil and the embedded permanent magnet. The coil‐generated torque τ_*coil*_ provides the transient driving moment required to overcome magnetic adhesion and deploy the side panels upward. Considering the actual thickness of the origami mechanism, the coil–magnet distance *d*′ is given by:

(4) $$\begin{array}{c} \;d^{\prime }=\sqrt{\frac{h^{2}}{4}-hl\sin\theta +4l^{2}\mathrm{si}n^{2}\frac{\theta }{2}} \end{array}$$

Accordingly, when the actuator starts from the flattened configuration, the magnitude of the initial coil‐induced torque can be written as:

(5) $$\begin{array}{c} \;\left|\tau_{coil}\left(0\right)\right|=\frac{24\mu_{0}\left|mm_{coil}\right|l}{\pi h^{4}} \end{array}$$

where *m*_coil_ is the equivalent magnetic dipole moment of the coil. The large initial torque generated at the flattened configuration provides the driving moment required to overcome magnetic adhesion and initiate side‐panel deployment, thereby enabling rapid transition from the free state to the constrained state.

Figure 3d presents the applied voltage and side‐panel angle over five consecutive locking–release cycles. The locking response time was measured from the onset of the forward pulse to attainment of the prescribed locking angle of 75°, and the releasing response time from the onset of the reverse pulse to return to the flattened state. Across the five uncycled actuators (i.e., those not subjected to the 1,000‐cycle durability protocol), each tested over five locking–release cycles, the locking and releasing response times were 102.8 ± 11.1 ms and 141.7 ± 4.7 ms, respectively (mean ± SD of device‐level averages; n = 5 actuators). The locked state required zero holding power. For use in virtual environments, however, the actuator must also remain stable in the free‐space state under arbitrary orientations. To this end, a small resting current was applied in the flattened state to provide weak static magnetic adhesion. Under a single‐cycle protocol of 1 s resting adhesion, 150 ms forward pulse, 1 s locked‐state holding, and 150 ms reverse pulse, the actuator consumed 444 mJ. As a wearable device in close contact with the skin, maintaining a safe temperature level is vital. Prior modeling work predicted that wearable‐device temperatures of approximately 34°C and above may negatively affect thermal comfort [47]. A long‐duration temperature test conducted under the above operating cycle showed that the coil temperature of the actuator eventually stabilized at 33.1°C, remaining below the thermal‐comfort reference (Figure 3e).

Finally, to comprehensively evaluate the mass‐efficient stiffness switching and load‐bearing performance of MagGami, we distinguish effective rotational stiffness from characteristic torque. The effective rotational‐stiffness ratio is defined as $\;R_{s}=\frac{K_{\mathrm{locked}}}{K_{\mathrm{free}}}\;$. *K*_**free**_ is the effective rotational stiffness in the free state, calculated as the slope of the line connecting the initial point and the point of maximum passive resistance within the prescribed angular range. *K*_**locked**_ is the effective rotational stiffness in the locked state, determined by least‐squares linear fitting of the loading branch of the torque–angle curve over the prescribed pre‐failure interval (Figure S3). Across the five uncycled actuators, *K*_**free**_ and *K*_**locked**_ were 0.198 ± 0.023 and 157.12 ± 17.77 N·mm·rad^−^^1^, respectively (mean ± SD; n = 5 actuators). The ratio *K*_**locked**_/*K*_**free**_ was calculated separately for each actuator and then averaged across the five actuators, giving 795.46 ± 61.71 (mean ± SD). Using an assembled actuator mass of 1.2 g (Figure S4), the corresponding module‐level SSD was (6.63 ± 0.51) × 10^5^ kg^−^^1^. The measurement and stiffness‐extraction procedures are described in the Methods, and individual‐actuator results and group summaries are provided in Table S2.

To separately characterize absolute load‐bearing capacity, *M*_**fail**_ denotes the maximum externally applied torque resisted by the mechanically interlocked structure before failure. Across the five uncycled actuators, *M*_**fail**_ was 76.25 ± 5.72 N·mm (mean ± SD; n = 5 actuators), corresponding to a resisted‐torque density of 63.54 ± 4.77 N·m·kg^−^^1^. Thus, *M*_**fail**_ represents the pre‐failure load‐bearing capacity of the interlocked structure rather than actuator‐generated torque or electromagnetic locking torque.

To assess the effects of repeated switching on actuator performance, we compared an uncycled reference group with an independent group characterized after 1,000 locking–release cycles (Table S2; n = 5 actuators per group). Relative to the mean of the uncycled group, the cycled group retained 90.36% of *K*_**locked**_ and 94.80% of *M*_**fail**_. Mean *K*_**free**_ decreased by 16.89%; because this reduction was greater than that in *K*_**locked**_, the mean device‐level stiffness ratio increased by 9.13%. Meanwhile, the mean locking and releasing response times increased by 0.97% and 1.21%, respectively. All five cycled actuators completed the protocol and remained operational before the final destructive test. These descriptive comparisons show that the actuators retained most of their locked‐state stiffness and pre‐failure load‐bearing capacity, while preserving a large stiffness contrast and exhibiting only small changes in switching time after 1,000 cycles under the tested conditions. Together, the large stiffness contrast, load‐bearing capacity, and retained performance after repeated switching provide the mechanical basis for the glove‐level evaluation in the following section.

### 2.4 Integrated 9‐DOF Haptic Glove Enhances Perceived Stiffness Through Joint‐Space Constraint Feedback

To translate the miniature MagGami modules into a functional wearable haptic interface, we integrated nine independently addressable actuators into a distributed 9‐DOF haptic glove spanning the thumb, index finger, and middle finger (Figure 4b; Figure S5). Specifically, three actuators were assigned to the thumb to constrain CMC flexion/extension‐related opposition as well as flexion of the MCP and IP joints, while the remaining six actuators were distributed across the MCP, PIP, and DIP joints of the index and middle fingers. The complete on‐body system, including the finger‐mounted actuator assembly and a forearm‐mounted control unit containing a rigid printed circuit board (PCB), heat‐dissipation components, a battery, an enclosure, and a fixation strap, weighs 78.36 g (Figure S6). The component‐level mass breakdown and measurement boundary are provided in the Methods. Together with the Unity3D rendering engine, optical hand tracking, an ESP32‐based low‐level controller, and a multichannel DRV8833 driver array, these modules formed a closed‐loop visuo‐haptic interaction system capable of mapping virtual contact states into distributed joint‐space constraints in real time (Figure 4a; Figures S7 and S8). Unlike fingertip‐based Cartesian‐space force‐feedback gloves, this architecture establishes a complementary multi‐node joint‐space interface that applies independently addressable virtual constraints to anatomically localized joints. The present system prioritizes lightweight, low‐profile binary constraint cues, and is not intended to match the continuous active‐force output or graded stiffness‐rendering capability available in many conventional force‐feedback systems.

**FIGURE 4:**
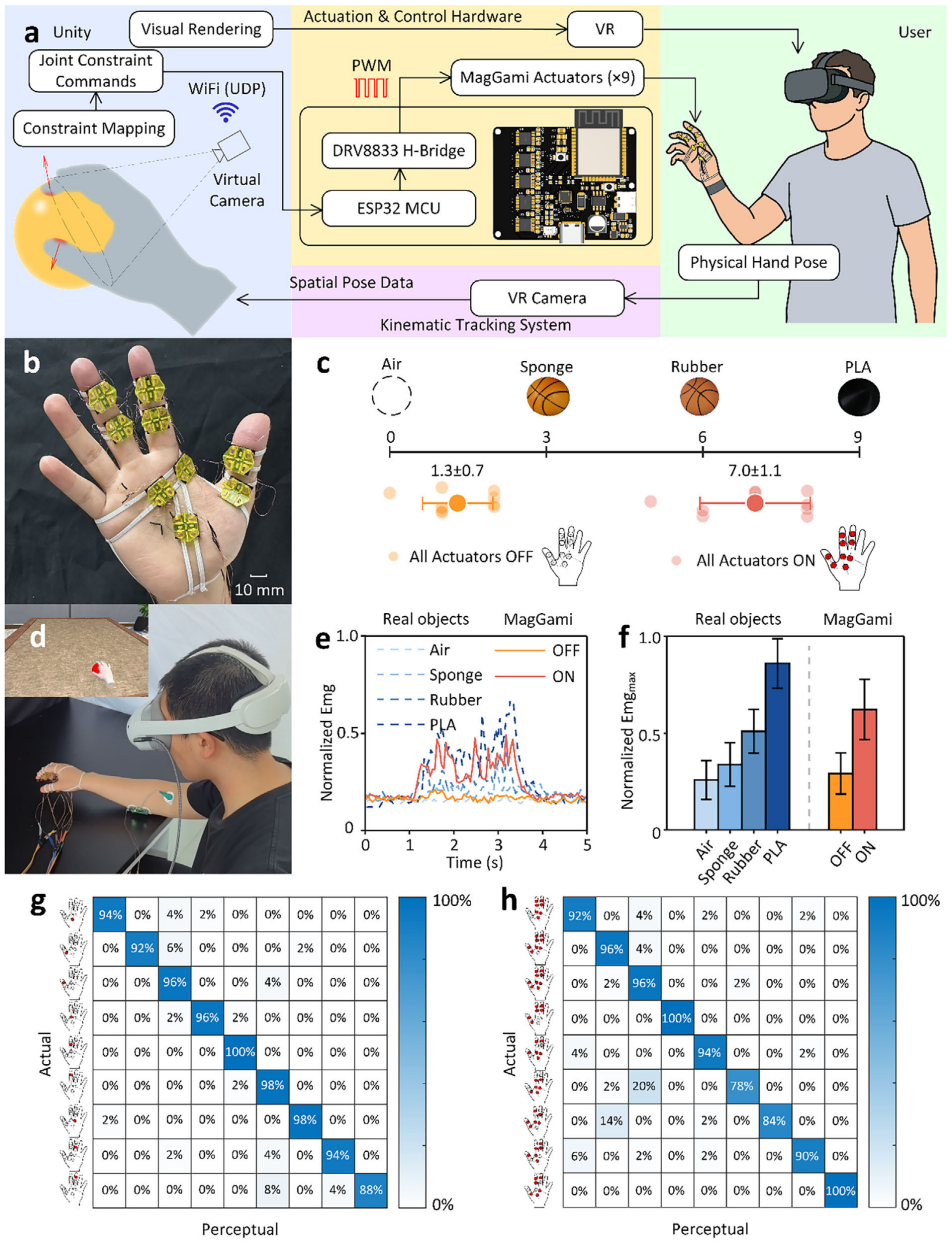
User studies of the MagGami‐based 9‐DOF haptic glove. a) Closed‐loop visuo‐haptic interaction architecture integrating virtual rendering, optical hand tracking, wireless communication, and distributed actuator control. b) Physical implementation of the MagGami glove with nine distributed actuators mounted across the thumb, index finger, and middle finger. Scale bar: 10 mm. c) Subjective stiffness ratings of the free and fully constrained virtual grasping conditions, compared with the anchored real‐object reference scale. Each virtual condition comprised five grasping trials per participant, and one subjective stiffness rating was recorded for each participant and condition. Summary markers and error bars indicate the mean ± SD across participant‐level ratings (n = 10). d) Experimental setup for immersive VR grasping and electromyography (EMG) recording. e) Representative EMG signals recorded during grasping of real objects and virtual objects rendered by the MagGami glove. f) Normalized maximum sEMG amplitudes for the four reference conditions and the two virtual conditions. For each participant and condition, the five normalized trial‐level maximum sEMG amplitudes were averaged; bars and error bars indicate the mean ± SD across participant‐level averages (n = 10). g, h) Confusion matrices for single‐joint and multi‐joint pattern recognition, respectively. Rows indicate actual patterns, and columns indicate participant‐reported patterns. Each matrix contains 450 trials (9 patterns × 10 participants × 5 repetitions), with 50 trials per target pattern. Values are row‐normalized percentages.

To quantify how joint‐space constraints influence perceived stiffness, 10 participants (see Methods for details) were recruited to compare the perceived stiffness of a glove‐rendered virtual sphere with that of real reference spheres of different stiffness levels. Three real reference spheres with a diameter of 6 cm, made of sponge, rubber, and rigid PLA, were selected as low‐, intermediate‐, and high‐stiffness anchors, respectively. In addition, virtual air grasping without wearing the glove was used as the zero‐stiffness reference condition. These four reference conditions were assigned to an anchored stiffness scale, with air, sponge, rubber, and PLA corresponding to scores of 0, 3, 6, and 9, respectively. Participants then grasped a visually identical virtual sphere rendered under two actuation states: an all‐actuators‐OFF state, corresponding to the free‐space condition, and an all‐actuators‐ON state, corresponding to a fully constrained grasp in which all nine relevant joints were locked. Participants rated the perceived stiffness of each virtual condition using this anchored reference scale.

The subjective ratings showed a clear separation between the two actuation states (Figure 4c). At the participant level, the all‐actuators‐OFF and all‐actuators‐ON conditions received ratings of 1.3 ± 0.7 and 7.0 ± 1.1, respectively (mean ± SD). An exact two‐sided Wilcoxon signed‐rank test confirmed that the ON condition was rated significantly higher than the OFF condition (p = 0.002, rank‐biserial r = 1.00), with all ten participants assigning a higher rating to the ON condition. These results place the OFF condition close to the air/sponge side of the scale, whereas the ON condition shifted perceived stiffness toward the high‐stiffness range represented by rubber and rigid PLA.

To establish physiological references across increasing levels of grasping resistance, we recorded sEMG signals under the air, sponge, rubber, and PLA reference conditions (Figure 4d; Figure S9). The normalized maximum sEMG amplitude increased progressively across these four conditions (Figure 4e,f). A one‐way repeated‐measures ANOVA revealed a significant effect of reference condition (F(3,27) = 247.87, p < 0.001, partial η^2^ = 0.965), and a linear contrast across the ordered reference conditions supported an overall increasing trend (p < 0.001).

During virtual grasping, the all‐actuators‐ON condition elicited a significantly higher normalized maximum sEMG amplitude than the all‐actuators‐OFF condition (0.622 ± 0.141 vs. 0.293 ± 0.109; mean ± SD across participant‐level averages, n = 10; paired t‐test: t(9) = 6.46, p < 0.001, Cohen's dz = 2.04).

Taken together, the subjective ratings and sEMG results demonstrate that switching the distributed actuators from the OFF state to the ON state produced a clear perceptual and physiological transition from compliant free‐space grasping to high‐resistance joint‐space constraint. MagGami is not intended to reproduce the continuous mechanical properties of physical materials. Instead, it provides salient distributed joint‐space constraints that strengthen the perception of virtual boundaries and resistant contact. These findings show that the glove provides perceptible joint‐constraint cues and increases perceived resistance during virtual grasping.

### 2.5 Distributed Joint‐Space Actuation Enables Discrimination of Localized Spatial Constraint Patterns

Beyond the whole‐hand ON/OFF evaluation, we next examined a specific capability enabled by distributed joint‐space actuation: the encoding and transmission of localized constraint patterns. To evaluate this capability, we conducted two additional discrimination experiments aimed at quantifying the independent perceptual addressability of individual actuator nodes and the recognizability of representative multi‐joint constraint patterns generated by coordinated activation of multiple nodes. The former examined whether individual joint‐level cues could be stably identified by users, whereas the latter evaluated whether the glove could convey structured information about where virtual motion was restricted, rather than merely a nonspecific sense of global resistance.

To quantitatively evaluate the perceptual addressability of individual actuator nodes, 10 participants (see Methods for details) were recruited to identify the activated joint among the nine distributed actuator locations. In each trial, only one actuator was activated, and participants were asked to report which joint on the thumb, index finger, or middle finger had been constrained (Figure S10A). The resulting confusion matrix exhibited strong diagonal dominance, with a participant‐level mean recognition accuracy of 95.1 ± 3.9% (mean ± SD, n = 10; Figure 4g), indicating that participants could reliably identify the activated joint among the nine distributed actuator nodes. Misclassifications were infrequent and were primarily concentrated between anatomically adjacent joints, particularly within the same finger. These errors may reflect biomechanical coupling or sensory overlap between adjacent joints; the present experiment did not isolate their sources. The high recognition performance demonstrates that the distributed MagGami array preserves excellent perceptual addressability after wearable integration, enabling individual joint constraints to be rendered as distinct and identifiable kinesthetic cues.

We next investigated whether the glove could convey more complex joint‐space constraint patterns when multiple actuators were activated simultaneously. To this end, nine representative multi‐joint actuation patterns were designed (Figure S10B). Some patterns differed in the extent of joint‐space constraint coverage, whereas others contained the same number of activated actuators and differed only in the spatial arrangement of the unconstrained joints across the thumb, index finger, and middle finger. Importantly, this design prevented trivial classification based on a single salient local cue and instead required participants to perceive the distributed organization of constraints across multiple joints.

The resulting confusion matrix for multi‐joint pattern discrimination again exhibited a pronounced diagonal structure, with a participant‐level mean recognition accuracy of 92.2 ± 4.6% (mean ± SD, n = 10; Figure 4h). Notably, several patterns containing the same number of activated joints but different spatial arrangements remained distinguishable. This supports the perceptual discriminability of spatially organized joint constraints, although the present experiment does not isolate spatial distribution from differences in the resulting resistance. In parallel, patterns with progressively increasing joint‐space coverage were also robustly recognized, suggesting that the glove can encode not only localized constraint gaps but also increasingly complete grasp‐related enclosure templates. Some of these patterns can be approximately associated with representative interaction states, such as localized grasping, single‐joint motion, and near‐complete grasping, corresponding to different spatial constraint configurations.

To examine whether recognition performance changed systematically over the five repetitions, trial‐level correctness was analyzed separately for the two tasks using binomial generalized linear mixed‐effects models. Repetition number was not significantly associated with recognition accuracy in either the single‐joint task (odds ratio = 0.953, 95% CI [0.703, 1.292], p = 0.757) or the multi‐joint task (odds ratio = 1.098, 95% CI [0.858, 1.404], p = 0.456). Thus, no detectable systematic repetition‐related improvement or decline was observed during the formal trials.

Taken together, these results demonstrate that the MagGami glove supports both independent joint‐level actuation and perceptually distinguishable multi‐joint spatial constraint encoding. Rather than generating only a global whole‐finger resistance, the distributed actuator array provides spatially organized constraint cues across the hand. This capability enables users to perceive where virtual motion is restricted and to distinguish different joint‐space contact patterns, offering a scalable route toward lightweight spatial constraint perception in immersive VR/AR environments.

## 3 Conclusion

In this work, we present MagGami, a miniaturized origami‐based electromagnetically switchable variable‐stiffness actuator for lightweight joint‐space kinesthetic feedback in immersive VR/AR interactions. As a complement to conventional Cartesian‐space force‐feedback approaches, the proposed distributed architecture applies binary constraint feedback directly at individual finger joints while maintaining a lightweight and low‐profile wearable form factor. The actuator combines origami geometric interlocking with an electromagnetic bistable mechanism, enabling rapid switching from a freely compliant state to a self‐locked high‐resistance state through transient current pulses. The resulting large stiffness contrast, ultrahigh stiffness‐switching density, and zero coil power for locked‐state holding address the critical force‐power‐volume trade‐off in wearable haptic interfaces.

At the system level, the 9‐DOF MagGami glove integrates optical hand tracking and VR rendering to generate localized joint‐space constraint cues. Importantly, the objective of the proposed system is not to fully reproduce the continuous mechanical behavior of physical objects. Instead, the joint‐space kinesthetic cues provided by MagGami are combined with visual information in the VR environment to enhance the perception of virtual spatial constraints and stiffness differences. User studies show that the fully constrained condition yields higher participant‐reported stiffness ratings and higher normalized maximum sEMG amplitudes than the free condition, while enabling reliable recognition of both single‐joint and multi‐joint constraint patterns. These results demonstrate that MagGami provides an effective hardware strategy for enhancing spatial constraint perception through lightweight, high‐contrast joint‐space force feedback, without requiring bulky external transmissions or continuous coil power for locked‐state holding. MagGami is therefore intended to complement rather than replace conventional Cartesian‐space force‐feedback gloves. The present study does not establish relative perceptual benefits over Cartesian‐space or less spatially distributed feedback. Future work will compare these approaches using common interaction tasks, while calibrating feedback output and explicitly characterizing differences in device mass, bandwidth, and control strategy. Future work will extend the current binary free/constrained switching at individual joints toward multi‐level or continuous stiffness modulation, enabling richer and more graded kinesthetic feedback. In addition, combining joint‐space kinesthetic constraints with cutaneous tactile feedback may further improve the richness and continuity of virtual interaction.

## 4 Methods

### 4.1 Fabrication of Actuators

The MagGami actuator was fabricated by combining high‐precision 3D printing with multistep micro‐assembly to ensure structural compactness and reliable electromagnetic coupling. The fabrication of each actuator unit involved three main steps (Figure S1).

Fabrication of origami panels and embedding of permanent magnets: The rigid origami panels were fabricated by projection micro‐stereolithography (PµSL) 3D printing (microArch S140, BMF) using a high‐resolution Tough photosensitive resin, providing a dimensional accuracy of 10 µm for the microscale hinges and magnet cavities. Permanent magnets (NdFeB N52, dimensions 3 mm × 1.5 mm × 0.5 mm) were then precisely inserted into the predesigned rectangular recesses on the two active side panels. Liquid UV‐curable adhesive (Samxya SD830HA, UV365–395 nm, 800 mJ/cm^2^) was dispensed into the interfacial gaps between the magnets and the resin matrix, and the assembly was subsequently cured in a UV curing system (Mercury Plus 3.0, ELEGOO, China) for 10 min to form a highly integrated monolithic structure.

Assembly of microscale hinges using carbon‐fiber shafts: To realize origami creases with both high kinematic compliance and mechanical robustness, carbon‐fiber filaments with a diameter of 0.3 mm were used as hinge shafts. Carbon fiber was selected owing to its high shear‐strength‐to‐weight ratio and minimal geometric footprint. The filaments were carefully threaded through aligned hinge channels in adjacent 3D‐printed panels. The unsealed ends of the channels were then sealed with UV adhesive and cured under ultraviolet light for 10 min, thereby fixing the rotational shafts in place and preventing axial displacement of the carbon‐fiber filaments.

Integration of the electromagnetic actuation layer: A rigid base plate was designed and fabricated according to the dimensions of the three fixed panels located on one side of the target finger‐joint crease. Circular copper coils (wire diameter 0.15 mm, outer diameter 6 mm, inner diameter 0.5 mm, height 1 mm) were arranged on the base plate such that their geometric centers were vertically aligned with the permanent magnets embedded in the active origami panels. Finally, 496 adhesive (LOCTITE, 20 g bottle, cyanoacrylate adhesive) was used to bond the copper coils, the rigid base plate, and the stationary side of the origami structure into an integrated electromagnetic locking variable‐stiffness actuator capable of independent deployment.

### 4.2 Control System and VR Integration

The hardware control system was implemented on a custom rigid PCB with a footprint of 40 mm × 50 mm and housed in a forearm‐mounted enclosure (Figures S7 and S8), enabling compact integration with the wearable haptic glove. The circuit integrated an ESP32‐WROOM‐32E microcontroller unit (MCU), a USB‐C communication and programming interface, onboard power‐management modules, and five DRV8833 dual H‐bridge drivers. Since each DRV8833 driver provides two independent bidirectional output channels, the circuit supports nine independent control outputs, which deliver forward and reverse pulse currents as well as maintenance currents to the MagGami actuators for on‐demand operation, thereby improving the compactness and wearability of the haptic glove. Copper‐foil heat spreaders were attached to the driver chips, and a miniature cooling fan was installed in the enclosure for thermal management.

The complete on‐body system weighed 78.36 g, comprising the finger‐mounted assembly (16.33 g; nine actuators, wiring, connectors, and finger‐fixation straps) and the fully assembled forearm control unit (62.03 g). The forearm unit included the populated PCB with copper‐foil heat spreaders (14.94 g), the 7.4 V lithium‐ion battery with its lead and connector (17.56 g), the enclosure (15.52 g), and the forearm strap and buckles (10.71 g). The combined additional mass of the cooling fan and assembly hardware was 3.30 g, determined by subtracting the sum of these four separately weighed components from the assembled‐unit mass. Masses were measured using a compact electronic balance with a resolution of 0.01 g (Figure S6).

The virtual reality interaction scenario was developed in Unity3D. During operation, Unity3D detected virtual grasping and release events and transmitted the corresponding spatial constraint commands wirelessly to the ESP32 MCU via UDP. Based on the received commands, the MCU generated independent pulse‐width modulation (PWM) control signals for the corresponding DRV8833 channels. Each channel delivered bidirectional transient pulse currents to the paired electromagnetic coil, thereby enabling reversible switching of the corresponding MagGami actuator between the unlocked and locked states. When the virtual hand grasped an object, a transient forward current was applied to the target coil to trigger origami interlocking and drive the actuator into the constrained state. Upon object release, a transient reverse current was applied to release the magnetic coupling and restore the origami mechanism to its flattened configuration, thereby returning the joint to the free state.

### 4.3 Mechanical Characterization and Cyclic Durability Testing

Ten independently fabricated MagGami actuators were assigned to two independent groups: an uncycled reference group (0 cycles; n = 5) and a cycled group (1,000 cycles; n = 5). Actuators in the cycled group underwent 1,000 consecutive locking–release cycles. Each cycle consisted of 1 s of resting adhesion, a 150 ms forward switching pulse, 1 s in the mechanically locked state, and a 150 ms reverse switching pulse, corresponding to a total cycle duration of 2.3 s.

The uncycled actuators were characterized directly, whereas the cycled actuators were characterized after completion of the 1,000‐cycle protocol. For each actuator, the locking and releasing response times were first measured using high‐speed video over five consecutive locking–release cycles. The five cycle‐level measurements were averaged to obtain one device‐level value for each switching direction. Free‐state mechanical characterization was then performed to determine *K*_**free**_. Finally, the actuator was switched to the locked state and subjected to a single monotonic loading‐to‐failure test. *K*_**locked**_ was obtained from the prescribed pre‐failure interval of the loading branch, and *M*_**fail**_ was defined as the maximum externally applied torque resisted before structural failure. This testing sequence ensured that the destructive locked‐state characterization did not affect the preceding measurements. Each actuator was treated as one independent sample, and all measurements were performed under identical loading and recording conditions. The resulting device‐level mechanical and response‐time measurements for the independent uncycled and cycled groups are presented in Table S2.

Torque–angle curves were obtained using a mechanical testing platform at a linear loading rate of 10 mm/min over a loading displacement of 10 mm. *K*_**free**_ was calculated as the secant stiffness between the initial point and the point of maximum passive resistance within the angular range converted from the displacement measurements. *K*_**locked**_ was determined by least‐squares linear fitting of the loading branch over the pre‐failure angular range (Figure S3). *M*_**fail**_ was defined as the maximum externally applied torque resisted before structural failure, which was identified by an abrupt change in the measured force upon reaching its peak. The stiffness ratio and module‐level SSD were calculated separately for each actuator before averaging within each group. Results are reported as mean ± SD across five independent actuators per group.

### 4.4 User Study Protocol

Ten healthy volunteers (7 male, 3 female, aged 22–28 years) participated in the study. All participants were right‐handed and reported no known neuromuscular disorders or hand injuries. The study was conducted with written informed consent from all participants and in accordance with the institutional requirements for human‐subject experiments. None had prior experience with the MagGami glove. Before the formal experiments, participants were familiarized with the VR environment and the operation of the haptic glove. During all VR‐based tasks, participants wore a VR headset, and visual instructions were presented directly in the virtual environment.

The user study consisted of two experiments designed to evaluate perceptual responses, neuromuscular activation, and the discriminability of distributed joint‐space constraint patterns.

Experiment 1: Virtual–Real Stiffness Perception

Participants first grasped four real reference objects—air, sponge, rubber, and PLA—to establish baseline sEMG signals. Each real‐object grasping condition was repeated five times per participant. These trials provided physiological baselines for different stiffness levels. Participants then wore the MagGami glove and VR headset to perform virtual grasping tasks under two device conditions: (i) all actuators deactivated (OFF condition) and (ii) all actuators activated (ON condition). For each participant and virtual condition, one subjective stiffness rating was recorded on the 0–9 scale, where 0 corresponded to free‐space grasping and 9 corresponded to the stiffest reference object. The rating was treated as the participant‐level observation for statistical analysis and was not averaged across repeated trials. Simultaneously, sEMG signals were recorded to compare neuromuscular responses elicited by the glove with those measured during real‐object grasping.

For each trial, we extracted the maximum ENV amplitude within the 5 s trial window. For each participant, the largest single‐trial maximum ENV amplitude across all 30 trials and six conditions was used as the participant‐specific normalization reference. Each trial‐level maximum ENV amplitude was divided by this reference, and the five normalized amplitudes were then averaged within each participant and condition to obtain one participant‐level value. Participant‐level OFF and ON subjective ratings were compared using an exact two‐sided Wilcoxon signed‐rank test, with rank‐biserial correlation reported as the effect size. The four reference sEMG conditions were analyzed using a one‐way repeated‐measures ANOVA, followed by a linear contrast across the ordered reference conditions. The sEMG amplitudes under the all‐actuators‐OFF and all‐actuators‐ON conditions were compared using a paired t‐test. Statistical analyses were performed using participant‐level condition averages, and results are reported as mean ± SD across participants (n = 10).

Experiment 2: Distributed Joint‐Space Constraint Discrimination

To evaluate the discriminability of distributed joint‐space constraint patterns, participants performed two tasks: single‐joint actuation and representative multi‐joint actuation patterns. Before formal testing, participants were first familiarized with all candidate patterns and the response procedure. For both single‐joint and multi‐joint tasks, each pattern was demonstrated once with its corresponding pattern number indicated on the reference panel. The patterns were then presented in a randomized order for practice, during which correctness feedback was provided. Formal testing began after participants confirmed that they understood the correspondence between the perceived patterns and their associated numbers. All familiarization and practice trials were excluded from the analysis. During the experiment, a reference panel illustrating all candidate patterns was continuously displayed in the VR environment to facilitate response reporting. However, the currently activated pattern was not indicated to the participant. For each participant and each task, a 45‐trial sequence containing five repetitions of each of the nine candidate patterns was generated and independently randomized by the program. The target pattern in each trial was then rendered by the glove through distributed joint‐level actuation. Participants identified the perceived pattern solely based on the haptic sensation generated by the glove and reported the corresponding pattern number using the reference panel. No correctness feedback was provided during the formal trials. All participants completed the single‐joint task before the multi‐joint task. For both single‐joint and multi‐joint tasks, this resulted in 450 recognition trials per task (9 patterns × 10 participants × 5 repetitions), totaling 900 trials. Recognition performance was quantified using confusion matrices. Participant‐level recognition accuracy was calculated separately for each task. To examine possible learning effects across the five formal repetitions, trial‐level correctness was analyzed separately for the single‐joint and multi‐joint tasks using binomial generalized linear mixed‐effects models, with repetition number entered as a fixed effect and participant entered as a random intercept. Repetition number was defined as the chronological occurrence number of each target pattern within the randomized trial sequence.

This protocol allowed systematic assessment of subjective stiffness perception, neuromuscular response, and multi‐joint constraint‐pattern discriminability under controlled and repeatable conditions.

## Author Contributions

T.J. and Z.N. contributed equally to this work. Yan Zhang and D.W. supervised the study. T.J. and Z.N. conceived the idea and directed the project. T.J. and Z.N. designed the MagGami actuator, developed the electromagnetically switchable origami interlocking mechanism, fabricated the devices, conducted the main experiments, analyzed the data, developed the theoretical model, and drafted the manuscript. Q.T. developed the VR environment. Z.W., Y.Q., G.Y., B.S., Yong Zhou, Yan Zhang, and D.W. reviewed and revised the manuscript.

## Funding

This work was supported by the Zhiyuan Scholar Program of Beijing Academy of Artificial Intelligence (BAAI) under Grant No. KH54‐9798‐01.

## Conflicts of Interest

The authors declare no conflicts of interest.

## Supporting information




**Supporting File 1**: advs78363‐sup‐0001‐SuppMat.docx.

**Supporting File 2**: advs78363‐sup‐0002‐SuppMat.zip.

## Data Availability

The data that support the findings of this study are available from the corresponding author upon reasonable request.
