# Supplementary material for: MagGami: An Electromagnetic Origami Actuator With High Stiffness‐Switching Density for Lightweight Joint‐Space Wearable Haptics
Source: Adv Sci (Weinh). 2026 Oct 20:e78363. Online ahead of print. doi: 10.1002/advs.78363 (PMC13683896; doi:10.1002/advs.78363)
Supplement: Supplementary file 1 [file ADVS-9999-e78363-s001.docx]

Supporting Information for

MagGami: An Electromagnetic Origami Actuator with High Stiffness-Switching Density for Lightweight Joint-Space Wearable Haptics

*Tianhao Jin, Zirui Niu, Qingyun Tang, Zemin Wang, Yu Qiao, Guocai Yao, Yong Zhou, Boxue Shan, Yan Zhang*, and Dangxiao Wang**

*Corresponding author. Email: b25078@buaa.edu.cn

hapticwang@buaa.edu.cn

**This PDF file includes:**

Note S1

Figures S1 to S10

Table S1 and S2

Movies legends S1 to S4

**Other Supporting Information for this manuscript includes the following:**

Movies S1 to S4

Note S1 Derivation of the magnetic locking force and electromagnetic switching torque

To analytically estimate the magnetic locking capability and electromagnetic switching behavior of MagGami, the embedded permanent magnets and the driving coil were modeled as ideal magnetic dipoles. As shown in Figure S2, the coordinate origin was set at the right rotation axis, with the $x$-axis pointing horizontally to the right and the $y$-axis pointing vertically upward. The distance between the two rotation axes was denoted as $a$, the distance from the magnet center to the rotation axis was denoted as $l$, and the thickness of the origami mechanism was denoted as $h$. Since the thickness direction of the mechanism is located below the rotation-axis plane, the magnet center has a vertical offset of $-h/2$ when $\theta=0$.

The center coordinates of the right and left permanent magnets can be written as

$$P_{2}=\left( l\cos\theta+\frac{h}{2}\sin\theta, l\sin\theta-\frac{h}{2}\cos\theta\right)$$

and

$$P_{1}=\left( -a-l\cos\theta-\frac{h}{2}\sin\theta, l\sin\theta-\frac{h}{2}\cos\theta\right)$$

respectively. Therefore, the distance between the two permanent magnets is

$$d=a+2l\cos\theta+h\sin\theta$$

Assuming that the magnetic dipole moment of a single permanent magnet is $m$, the magnetic dipole moment vectors of the left and right magnets are expressed as

$$\mathbf{m}_{1}=m\left( \sin\theta,cos\theta\right)$$

and

$$\mathbf{m}_{2}=m\left( \sin\theta,-cos\theta\right)$$

respectively. According to the magnetic dipole interaction model, the magnetic potential energy between the two permanent magnets is

$$U=-\frac{\mu_{0}}{4\pi d^{3}}\left[ \mathbf{m}_{1}\cdot\mathbf{m}_{2}-3\left( \mathbf{m}_{1}\cdot{\hat{\mathbf{r}}}_{12} \right)\left( \mathbf{m}_{2}\cdot{\hat{\mathbf{r}}}_{12} \right) \right]$$

where ${\hat{\mathbf{r}}}_{12}$ is the unit vector from the left magnet to the right magnet. Since the two magnets are horizontally aligned,

$${\hat{\mathbf{r}}}_{12}=\left( 1,0 \right)$$

Thus,

$$\mathbf{m}_{1}\cdot\mathbf{m}_{2}=m^{2}\left( \sin^{2}\theta-\cos^{2}\theta\right)$$

$$\mathbf{m}_{1}\cdot{\hat{\mathbf{r}}}_{12}=m\sin\theta$$

$$\mathbf{m}_{2}\cdot{\hat{\mathbf{r}}}_{12}=m\sin\theta$$

Substituting these terms into the dipole interaction equation gives the magnetic potential energy in the locked state as

$$U_{lock}=\frac{\mu_{0}m^{2}}{4\pi d^{3}}\left( 1+\sin^{2}\theta\right)$$

namely,

$$U_{lock}=\frac{\mu_{0}m^{2}}{4\pi\left( a+2l\cos\theta+h\sin\theta\right)^{3}}\left( 1+\sin^{2}\theta\right)$$

The horizontal attractive force $F_{lock}$ is obtained from the negative derivative of the potential energy with respect to the spatial distance $d$:

$$F_{lock}=-\frac{\partial U_{lock}}{\partial d}$$

Therefore,

$$F_{lock}=-\frac{3\mu_{0}m^{2}}{4\pi d^{4}}\left( 1+\sin^{2}\theta\right)$$

namely,

$$F_{lock}=-\frac{3\mu_{0}m^{2}}{4\pi\left( a+2l\cos\theta+h\sin\theta\right)^{4}}\left( 1+\sin^{2}\theta\right)$$

The locking torque generated by the magnetic attraction can be obtained from the negative derivative of the potential energy with respect to the rotation angle $\theta$:

$$\tau_{lock}=-\frac{\partial U_{lock}}{\partial\theta}$$

Since

$$\frac{dd}{d\theta}=-2l\sin\theta+h\cos\theta$$

the locking torque is

$$\tau_{lock}=-\frac{\mu_{0}m^{2}}{4\pi}\left[ \frac{2\sin\theta\cos\theta}{d^{3}}+\frac{3\left( 1+\sin^{2}\theta\right)\left( 2l\sin\theta-h\cos\theta\right)}{d^{4}} \right]$$

Substituting $d=a+2l\cos\theta+h\sin\theta$, we obtain

$$\tau_{lock}=-\frac{\mu_{0}m^{2}}{4\pi}\left[ \frac{2\sin\theta\cos\theta}{\left( a+2l\cos\theta+h\sin\theta\right)^{3}}+\frac{3\left( 1+\sin^{2}\theta\right)\left( 2l\sin\theta-h\cos\theta\right)}{\left( a+2l\cos\theta+h\sin\theta\right)^{4}} \right]$$

The electromagnetic switching process was further analyzed by considering the interaction between the coil and the permanent magnet. The equivalent magnetic dipole moment of the coil was denoted as $m_{coil}$, and the equivalent dipole point of the coil was set as

$$C=\left( l,0 \right)$$

The distance between the coil and the magnet is then

$$d'=\sqrt{\left( l\cos\theta+\frac{h}{2}\sin\theta-l \right)^{2}+\left( l\sin\theta-\frac{h}{2}\cos\theta\right)^{2}}$$

which can be simplified as

$$d'=\sqrt{\frac{h^{2}}{4}-hl\sin\theta+4l^{2}\sin^{2}\frac{\theta}{2}}$$

According to the magnetic dipole interaction model, the potential energy between the energized coil and the permanent magnet can be written as

$$U_{coil}=\frac{\mu_{0}mm_{coil}}{8\pi}\frac{Q}{d'^{5}}$$

where

$$Q=h^{2}\cos\theta-3hl\sin\theta-\frac{hl}{2}\sin2\theta-4l^{2}\cos\theta-l^{2}\cos2\theta+5l^{2}$$

The torque generated by the coil is similarly obtained from the negative derivative of the potential energy with respect to $\theta$:

$$\tau_{coil}=-\frac{\partial U_{coil}}{\partial\theta}$$

For simplification, we define

$$Q'=-h^{2}\sin\theta-3hl\cos\theta-hl\cos2\theta+4l^{2}\sin\theta+2l^{2}\sin2\theta$$

Since

$$\frac{d\left( d'^{2} \right)}{d\theta}=-hl\cos\theta+2l^{2}\sin\theta$$

the coil-induced torque can be obtained as

$$\tau_{coil}=\frac{\mu_{0}mm_{coil}}{8\pi}\frac{5Q\left( l^{2}\sin\theta-\frac{hl}{2}\cos\theta\right)-Q'd'^{2}}{d'^{7}}$$

namely,

$$\tau_{coil}=\frac{\mu_{0}mm_{coil}}{8\pi}\frac{5Q\left( l^{2}\sin\theta-\frac{hl}{2}\cos\theta\right)-Q'\left( \frac{h^{2}}{4}-hl\sin\theta+4l^{2}\sin^{2}\frac{\theta}{2} \right)}{\left( \sqrt{\frac{h^{2}}{4}-hl\sin\theta+4l^{2}\sin^{2}\frac{\theta}{2}} \right)^{7}}$$

When $\theta$ approaches 0, the coil–magnet distance approaches $h/2$, indicating that a finite gap determined by the mechanism thickness remains between the coil and the magnet. Therefore, the coil-induced switching torque does not exhibit a mathematical singularity. The magnitude of the initial switching torque under the finite-thickness gap is

$$\left| \tau_{coil}\left( 0 \right) \right|=\frac{24\mu_{0}\left| mm_{coil} \right|l}{\pi h^{4}}$$

This finite but large initial torque explains why, at the beginning of actuation in the flattened state, a small transient current pulse can generate a strong burst torque to rapidly flip up the origami side panels and trigger the mechanical locking transition.

Figure S1. Fabrication process of the MagGami origami mechanism. The rigid origami panels were first fabricated by high-resolution 3D printing with predesigned magnet cavities and hinge channels. Permanent magnets were then inserted into the rectangular recesses of the active side panels and fixed using UV-curable adhesive. Microscale carbon fiber tows were threaded through the aligned hinge channels to serve as rotational shafts for the origami creases. The hinge openings were subsequently sealed with UV-curable adhesive and cured under UV irradiation to prevent axial displacement of the carbon fiber shafts. After multistep assembly and curing, the integrated origami mechanism was obtained, providing compact foldability, reliable hinge motion, and mechanical robustness for electromagnetic locking actuation.

Figure S2. Geometric model and magnetic dipole approximation for the finite-thickness MagGami actuator. The coordinate origin is set at the right rotation axis. The permanent magnets embedded in the origami side panels and the driving coils are simplified as magnetic dipoles to derive the magnetic locking force, locking torque, and coil-induced switching torque.

Figure S3. Illustration of effective rotational-stiffness extraction from free- and locked-state torque–angle curves. Solid red and gray curves show the locked and free states, respectively. The magenta dashed line shows the least-squares linear fit to the pre-failure locked-state loading branch used to determine *K*_locked_. The blue dashed line shows the free-state secant used to determine *K*_free_. Angles are plotted in degrees, whereas stiffnesses are reported per radian. Individual-actuator results and group summaries are provided in Table S2.

Figure S4. Photograph of an assembled MagGami actuator being weighed on an electronic balance. The actuator mass, including the support structure, was 1.20 g.

Figure S5. Photographs of the wearable MagGami glove. (A) Palmar view showing the nine finger-mounted actuators and fixation straps. (B) Dorsal view showing the wiring arrangement and forearm-mounted control unit.

Figure S6. Mass measurements of the MagGami wearable system and its components. (A) Finger-mounted actuator assembly, 16.33 g. (B) Fully assembled forearm control unit, 62.03 g. (C) Populated PCB with copper-foil heat spreaders, 14.94 g. (D) Enclosure, 15.52 g. (E) Battery with its lead and connector, 17.56 g. (F) Forearm strap and buckles, 10.71 g. The complete on-body mass was 78.36 g, calculated as (A) + (B). The components in (C)–(F), cooling fan, and assembly hardware are included in (B).

Figure S7. Photographs of the custom rigid printed circuit board (PCB). (A) Top view. (B) Front view. A caliper is included as a dimensional reference.

Figure S8. Schematic of the custom PCB-based control circuit for the MagGami actuator array. The circuit integrates an ESP32 microcontroller, a USB-C communication and programming interface, power-management components, and five DRV8833 dual H-bridge drivers. Bidirectional outputs drive the nine actuator coils, and an additional driver output is connected to the cooling fan.

Figure S9. Photograph of the sEMG recording process during grasping of a rubber ball.

Figure S10. Visual reference panels for the joint-state discrimination experiments in the VR environment. (A) Nine single-joint actuation patterns used to evaluate the localization accuracy of independently activated MagGami actuators. (B) Nine representative multi-joint actuation patterns used to evaluate the discriminability of distributed joint-space constraint configurations. Red filled circles denote activated actuators, and hollow circles denote inactive actuators.
Table S1. Performance comparison of representative force feedback gloves & actuators

| **Literature** | **Actuation Type** | **Total Mass (g)** | **Actuator Mass (g)** | **Max Output Torque / Force** | **Stiffness Ratio (Rs)** | **Response Time (ms)** | **Power** |
| --- | --- | --- | --- | --- | --- | --- | --- |
| This work | Smart Material / Origami electromagnetic interlocking | 78.36 | 1.2 (actuator) | 76.25 N·mm | 795.46 | 102.8 | 444 mJ/cycle |
| [1] | Pneumatic / PneuClutch | 283 | N/A | 22.41 N (max resistive) / 3.23 N (indentation) | ~3.06 | 18 | 1.12 W (avg.) |
| [2] | Smart Material / SMA | 490 | N/A | 14.3 N (max fingertip force) | NR | ~700 | NR |
| [3] | Pneumatic / Layer jamming | 299.11 | N/A | 11.95 N (3-point bending) | ~26.3 | 43 | NR |
| [4] | Smart Material / mSMA auxetic structure | 80  (estimated) | <0.005 (mSMA wire only) | 1.8 N (mSMA force) | ~5.3 | <100–>5000 | NR |
| [5] | Motor / Link-bar exoskeleton | 300  (estimated) | N/A | ~4.9 N (peak fingertip force) | NR | ~33 (30 Hz update) | NR |
| [6] | Smart Material / MR brake | 230  (estimated) | N/A | 9.86 N (fingertip force) | NR | 28 | NR |
| [7] | Motor / DC motors | 230.1 | N/A | ~1.5 N (matched tension) | NR | NR | NR |
| [8] | Pneumatic / Pneumatic artificial muscle (McKibben) | 600  (estimated) | <2 (per muscle) | ~8 N (push) / ~4 N (pull) | NR | ~100 | NR |
| [9] | Pneumatic / Layer jamming | 300  (estimated) | N/A | 77.0 N·mm (max torque) | ~2.6 | 200–300 | NR |
| [10] | Smart Material / Variable-stiffness rods | 100  (estimated) | N/A | 73.785 N·mm⁻¹ (max effective stiffness) | ~23.26 | ~31500 (heating) | NR |

Note: System mass and actuator/component mass are reported separately, with estimated and component-only values explicitly identified. Figure 1c compares stiffness ratio and inverse complete-system mass as two independent dimensions, rather than presenting a system-level SSD comparison. No cross-study module-level SSD values are reported in this table. Included system components may differ between studies. NR denotes not reported, and N/A denotes unavailable or non-comparable actuator-module mass data.

Table S2. Mechanical and switching performance of MagGami actuators

**Group summaries**

| **Performance metric** | **Unit** | **Uncycled group Mean ± SD** | **1,000-cycle group Mean ± SD** | **Mean change (%)** |
| --- | --- | --- | --- | --- |
| Free-state stiffness, K_free | N·mm·rad⁻¹ | 0.198 ± 0.023 | 0.165 ± 0.020 | -16.89% |
| Locked-state stiffness, K_locked | N·mm·rad⁻¹ | 157.12 ± 17.77 | 141.97 ± 10.07 | -9.64% |
| Stiffness ratio, R_s | Dimensionless | 795.46 ± 61.71 | 868.06 ± 76.72 | +9.13% |
| Maximum resisted torque, M_fail | N·mm | 76.25 ± 5.72 | 72.28 ± 12.35 | -5.20% |
| Locking response time | ms | 102.8 ± 11.1 | 103.8 ± 10.8 | +0.97% |
| Releasing response time | ms | 141.7 ± 4.7 | 143.4 ± 5.2 | +1.21% |

**Individual actuator data**

| **Actuator** | **K_free (N·mm·rad⁻¹)** | **K_locked (N·mm·rad⁻¹)** | **R_s** | **M_fail (N·mm)** | **Locking response time (ms; mean ± SD)** | **Releasing response time (ms; mean ± SD)** |
| --- | --- | --- | --- | --- | --- | --- |
| U1 | 0.162 | 136.87 | 844.58 | 84.86 | 107.3 ± 8.2 | 146.0 ± 8.3 |
| U2 | 0.200 | 140.27 | 700.74 | 74.63 | 104.0 ± 11.3 | 140.0 ± 7.6 |
| U3 | 0.226 | 177.93 | 787.56 | 69.12 | 113.2 ± 5.4 | 147.1 ± 8.8 |
| U4 | 0.208 | 163.86 | 787.88 | 75.07 | 84.0 ± 8.5 | 139.6 ± 4.8 |
| U5 | 0.195 | 166.66 | 856.53 | 77.57 | 105.7 ± 4.9 | 135.8 ± 7.5 |
| C1 | 0.148 | 131.89 | 890.04 | 79.79 | 113.6 ± 2.3 | 147.5 ± 9.8 |
| C2 | 0.197 | 150.40 | 761.94 | 51.45 | 90.3 ± 7.7 | 140.0 ± 5.4 |
| C3 | 0.156 | 130.35 | 835.23 | 71.74 | 108.2 ± 4.8 | 138.8 ± 3.7 |
| C4 | 0.151 | 146.37 | 970.60 | 82.61 | 112.8 ± 2.8 | 150.4 ± 7.2 |
| C5 | 0.171 | 150.85 | 882.51 | 75.83 | 94.4 ± 1.9 | 140.4 ± 1.9 |

a) Each group comprised five independently fabricated actuators. Group summaries are mean ± SD across five device-level values; response-time summaries use each actuator’s five-cycle mean. The groups were independent, and no pairing between actuators was assumed.

b) U1–U5 and C1–C5 denote uncycled and cycled actuators, respectively. Individual response-time values are mean ± SD across five locking–release cycles per actuator.

c) Percentage change was calculated as (cycled-group mean − uncycled-group mean)/uncycled-group mean × 100%, using unrounded data.

Movie S1.

Mechanical stiffness testing of the MagGami actuator.

Movie S2.

Pulse-driven switching response and thermal stability of the MagGami actuator.

Movie S3.

Wearable demonstration of compliant and constrained finger motion.

Movie S4.

VR-based free-space and constrained-space grasping with EMG recording.

**References**

[1] Qi J, Gao F, Sun G, et al. HaptGlove—untethered pneumatic glove for multimode haptic feedback in reality–virtuality continuum[J]. Advanced Science, 2023, 10(25): 2301044.
[2] Sui M, Ouyang Y, Jin H, et al. A soft-packaged and portable rehabilitation glove capable of closed-loop fine motor skills[J]. Nature Machine Intelligence, 2023, 5(10): 1149-1160.
[3] Wang Z, Zhou X, Zhou Z, et al. MateJam: Multi-material teeth-clutching layer jamming actuation for soft haptic glove[J]. IEEE Transactions on Haptics, 2023, 16(2): 276-286.
[4] Oh S, Jang J H, Ji W, et al. Embodied Auxetic Intelligence in a Glove‐Type Wearable Haptic Interface Connecting Humans to Robots and the Metaverse[J]. Advanced Functional Materials, 2025, 35(34): 2502222.
[5] Friston S, Griffith E, Swapp D, et al. Position-based control of under-constrained haptics: A system for the dexmo glove[J]. IEEE Robotics and Automation Letters, 2019, 4(4): 3497-3504.
[6] Chen D, Ni H, Lou J, et al. Design and evaluation of a compact force feedback glove for virtual reality applications[J]. Sensors and Actuators A: Physical, 2026: 117514.
[7] Baik S, Park S, Park J. Haptic glove using tendon-driven soft robotic mechanism[J]. Frontiers in bioengineering and biotechnology, 2020, 8: 541105.
[8] Takahashi N, Takahashi H, Koike H. Soft exoskeleton glove enabling force feedback for human-like finger posture control with 20 degrees of freedom[C]//2019 IEEE World Haptics Conference (WHC). IEEE, 2019: 217-222.
[9] Zhang Y, Wang D, Wang Z, et al. Passive force-feedback gloves with joint-based variable impedance using layer jamming[J]. IEEE Transactions on Haptics, 2019, 12(3): 269-280.
[10] Yang H, Patel D K, Johnson T, et al. A compliant metastructure design with reconfigurability up to six degrees of freedom[J]. Nature Communications, 2025, 16(1): 719.
